# Shaping caustics into propagation-invariant light

**DOI:** 10.1038/s41467-020-17439-3

**Published:** 2020-07-17

**Authors:** Alessandro Zannotti, Cornelia Denz, Miguel A. Alonso, Mark R. Dennis

**Affiliations:** 1 Institute of Applied Physics and Center for Nonlinear Science (CeNoS), University of Muenster, Muenster, 48149 Germany; 2 Aix Marseille University, CNRS, Centrale Marseille, Institut Fresnel, UMR 7249, Marseille, 13013 France; 3 The Institute of Optics, University of Rochester, Rochester, NY 14627 USA; 4 School of Physics and Astronomy, University of Birmingham, Birmingham, B15 2TT UK; 5 HH Wills Physics Laboratory, University of Bristol, Bristol, BS8 1TL UK

**Keywords:** Optical materials and structures, Optical physics, Optical physics

## Abstract

Structured light has revolutionized optical particle manipulation, nano-scaled material processing, and high-resolution imaging. In particular, propagation-invariant light fields such as Bessel, Airy, or Mathieu beams show high robustness and have a self-healing nature. To generalize such beneficial features, these light fields can be understood in terms of caustics. However, only simple caustics have found applications in material processing, optical trapping, or cell microscopy. Thus, these technologies would greatly benefit from methods to engineer arbitrary intensity shapes well beyond the standard families of caustics. We introduce a general approach to arbitrarily shape propagation-invariant beams by smart beam design based on caustics. We develop two complementary methods, and demonstrate various propagation-invariant beams experimentally, ranging from simple geometric shapes to complex image configurations such as words. Our approach generalizes caustic light from the currently known small subset to a complete set of tailored propagation-invariant caustics with intensities concentrated around any desired curve.

## Abstract (web summary)

Propagation-invariant beams have been up to now only available in a few realizations or for specific applications. Here, the authors demonstrate two methods for generating propagation-invariant beams with fully customizable intensity profiles in a comprehensive way inspired by geometrical optics.

## Introduction

The field of structured light has grown significantly since the early studies of Laguerre- and Hermite-Gaussian modes in laser cavities^1,2^. This growth stemmed both from increased theoretical understanding and from the advent of new optical devices such as spatial light modulators (SLMs). This area of research has now transcended optics, leading to a range of applications in fundamental physics^3^, telecommunications^4–6^, security^7–9^, micromachining^10,11^, imaging^12–15^, and the manipulation of cells and microorganisms^16,17^. In particular, the fact that phase-structured light carries orbital angular momentum (OAM)^18^ allows the exertion not only of forces but also of torques onto atoms, bacteria, or micromachines^1,2^.

A class of structured optical field that has received considerable attention is that of the so-called propagation-invariant or non-diffracting beams, whose transverse intensity distribution remains essentially invariant over a significant propagation distance^19^. Propagation-invariant beams have a transverse angular spectrum confined to a ring^20^ whose radius is proportional to their numerical aperture. The best-known examples are Bessel^21,22^, Mathieu^19,23,24^, and Weber^19,23,25^ beams, which are described by closed-form expressions that are separable in polar, elliptic and parabolic coordinates, respectively. The intensity maxima of these beams are therefore localized around the corresponding conic section shapes, characterized by their caustics^24,26–28^. Some of these propagation-invariant beams have been used in advanced optical trapping setups^16,17,29^, imaging with high resolution^14,15^, and ultrafast nanoscale material processing with high aspect ratios^10,11,30^. However, having only a limited set of beams of this type restricts significantly their usefulness. All the applications mentioned earlier would greatly benefit from the ability to tailor the shapes of propagation-invariant beams for the purpose at hand.

The achievement of our work is propagation-invariant beams with customizable intensity profiles. To design the respective beams, we develop two methods for shaping the corresponding caustics. These methods are illustrated with the experimental implementation of beams whose intensity features trace a range of geometrical shapes as well as more complex patterns such as words, which remain essentially invariant over a significant propagation distance.

## Results

### Caustics in propagation-invariant beams

The conceptual basis for this work is the relation between these wave solutions and the simpler ray model, for which the intensity maxima follow the shapes of the caustics, which are the envelopes of the two-parameter family of rays associated with the field^31^. Within the ray picture, propagation invariance requires that all rays travel at the same angle *θ* with respect to the propagation direction (chosen as the *z* axis), and that the two-parameter ray family is composed of one-parameter subfamilies of parallel rays constrained to planes parallel to *z*. This structure guarantees that the caustics themselves are invariant in *z*, since they correspond to the envelopes of these planes. The fact that all rays have equal angle with respect to the *z* axis implies that the transverse Fourier spectrum of the wave field is restricted to a ring^19^ (whose radius is ${k}_{\perp }=k\sin \theta$, where *k* is the wavenumber). The azimuthal angle *ϕ* of each subfamily of rays indicates the point along the ring to which they correspond.

The mathematical correspondence between the ring’s amplitude and phase distributions, *A*(*ϕ*) and Φ(*ϕ*), and the beam’s transverse field is given by Whittaker’s integral^19,20^:

1 $$\psi ({\bf{r}})=\oint A(\phi )\exp \left[{\rm{i}}\Phi (\phi )+{\rm{i}}{k}_{\perp }{\bf{r}}\cdot {\bf{u}}(\phi )\right]{\rm{d}}\phi ,$$

where **r** = (*x*, *y*) is the transverse position vector and ${\bf{u}}(\phi )=(\cos \phi ,\ \sin \phi )$ is a unit vector that indicates the transverse direction of the corresponding rays and the planes that embed them. The connection with ray optics and caustics results from applying the method of stationary phase^31^ to Eq. (1). As shown in the ‘Methods’ section, this leads to the following relation in terms of the parametrized caustic shape **r**_c_(*ϕ*):

2 $${{\bf{r}}}_{{\rm{c}}}(\phi )=\frac{1}{{k}_{\perp }}\big[{\Phi }^{{\prime\prime} }(\phi ){\bf{u}}(\phi )-{\Phi }^{\prime}(\phi ){{\bf{u}}}^{\prime}(\phi )\big].$$

By choosing *A* and Φ appropriately, different caustic shapes can be achieved. The beam’s intensity maxima are localized within the vicinity of these caustics. For example, Bessel beams (for which *A* is constant and Φ(*ϕ*) = *ℓϕ* for integer *ℓ*) have caustics with circular (or punctual, for *ℓ* = 0) cross-sections^28,32–34^, while Mathieu beams have caustics with elliptic or hyperbolic cross-sections^24^.

**Fig. 1:**
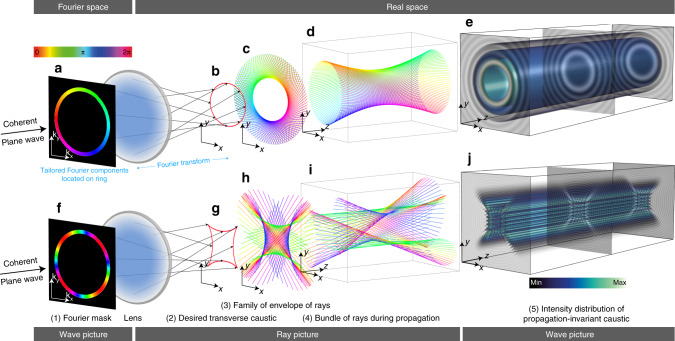
Caustics in propagation-invariant beams. **a**–**e** A Bessel beam *J*_1_ with OAM of charge *ℓ* = 1 has a circular caustic. **a** Fourier phase pattern, with azimuthally linearly increasing phase Φ = *ℓϕ*, confined to an infinitesimal thin ring. Fourier transform by a lens. Fourier wavefront shaping determines the direction of the propagating rays, and the caustic (**b**) forms in real space as the envelope of the rays in a transverse plane (**c**). A subset of the rays, each coming from a specific point on the ring, is shown in (**d**). The complete family of rays is composed of a continuum of bundles like the one shown in **d** but displaced in *z*. The corresponding field has a propagation-invariant transverse intensity (**e**) with a pronounced central ring according to the circular caustic. **f**–**j** A more complex phase function $\Phi =\ell \phi -q/2\sin (2\phi )$ yields an astroid caustic, which includes four cusp caustics.

  Figure 1a–e illustrates some of these ideas for the simple example of a Bessel beam with topological charge *ℓ* = 1. The transition between the Fourier plane in Fig. 1a and the physical space in Fig. 1b–e is implemented experimentally with a converging lens placed one focal length away from the Fourier plane. The cross-sections of the planes containing the rays shown in Fig. 1c form the circular caustic shown in Fig. 1b. The 3D configuration of some of these rays (one from each subfamily) is also shown in Fig. 1d; the complete two-parameter family of rays includes the subset shown as well as replicas displaced in *z* (see Supplementary Note 1 for a discussion of the whole ray family). Note that the rays are color coded (by hue) to show the corresponding phase at the ring. For visual convenience the accumulation of phase due to propagation is factored out. Finally, the 3D intensity distribution for this Bessel beam, dominated by the innermost intensity circle, is also shown in Fig. 1e. Figure 1f–j shows similar information for a more complicated beam with $\Phi (\phi )=-2.5\sin (2\phi )$. This phase distribution leads to a propagation-invariant astroid caustic that exhibits four cusps (see ‘Methods’ section for an explanation of the origin of these cusps). More information regarding the visualization of the rays is provided in Supplementary Note 1.

### Solving the inverse problem using the differential Eq. (2)

**Fig. 2:**
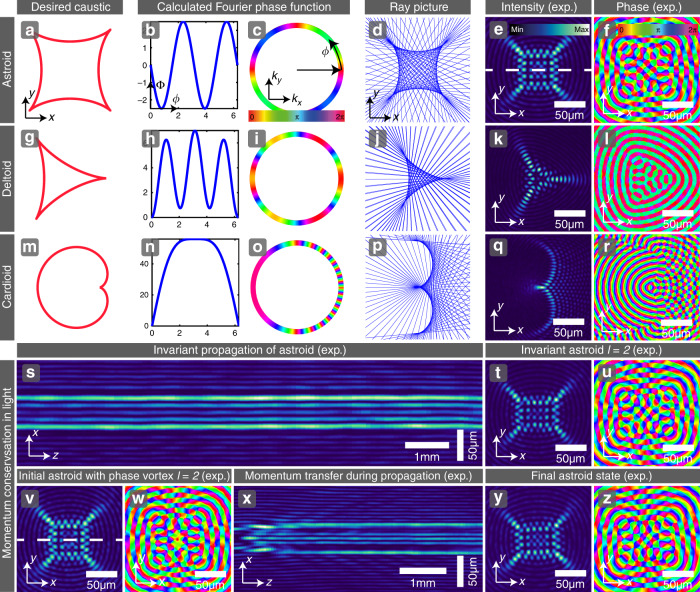
Engineering desired caustics in light, demonstrating their propagation invariance and momentum conservation. **a**–**f** Astroid. **a** Desired caustic. **b** 1D phase function. **c** Phase function mapped onto 2D Fourier ring. **d** Transverse projection of the rays. **e**, **f** Experimentally measured transverse intensity and phase. **g**–**l** Deltoid. **m**–**r** Cardioid. **s** Invariant propagation of the astroid from image (**e**), *xz*-cross-section through the intensity volume. **t**, **u** Invariant astroid with charge *ℓ* *=* 2. **v**–**z** Self-healing/momentum transfer in astroid: **v**, **w** Diffractive astroid, initial field with phase vortex of charge *ℓ* = 2. **x** Propagation showing diffraction and transfer to final OAM state. **y**, **z** Momentum transfer to final astroid state same as (**e**, **f**), which then propagates invariantly.

Our goal, however, is to solve the inverse problem: find the phase distribution Φ that produces any desired propagation-invariant caustic. The first approach is the solution of Eq. (2) for Φ(*ϕ*) for a given **r**_c_(*ϕ*). For simplicity we keep the amplitude *A*(*ϕ*) constant. The details of this approach are given in the ‘Methods’ section, and some results are shown in Fig. 2 for simple caustic configurations. For example, solving the differential equation for the astroid caustic shown in Fig. 2a gives the sinusoidal phase function $\Phi (\phi )=\ell \phi -q/2\ \sin (2\phi )$ shown in Fig. 2b, with $q\in {\mathbb{R}}$, which is mapped mod 2*π* on the Fourier ring shown in Fig. 2c. (Note that this astroid can carry OAM with topological charge *ℓ*.) From the Fourier phase function, we calculate the transverse ray picture of the beam shown in Fig. 2d. The resulting experimentally measured transverse intensity and phase distributions, shown in Fig. 2e, f, respectively (for *q* = 5), present a square lattice at the center, surrounded by several rings. This beam interpolates between Bessel beams (*q* = 0) and four interfering plane waves (*q* → *∞*): we call this light field a Bessel-lattice beam ${\psi }_{\ell ,q}^{{\rm{BL}}}$. Figure 2g–l, m–r shows the corresponding results for deltoid and cardioid caustics, respectively. We use a cw laser beam with wavelength *λ*_0_ = 2*π*/*k*_0_ = 532 nm, shaped by a SLM, for their experimental realization. All beams demonstrated here have the same real space structure size *a* = 2*π*/*k*_⊥_ = 15 μm related to the propagation constant *k*_*z*_ via ${k}_{0}^{2}={k}_{\perp }^{2}+{k}_{z}^{2}$. Figure 2s presents the experimental verification of the propagation invariance of the astroid caustic, by showing a *xz*-cross-section of the measured intensity volume (corresponding to the white dashed line in Fig. 2e). This Bessel-lattice beam with charge *ℓ* = 0 propagates for one Rayleigh length *z*_*e*_ = 2*k*_0_*a*^2^ = 5.32 mm without significantly changing its transverse intensity distribution. In general, all propagation-invariant beams realizable with our method have standard properties with respect to their diffraction length: theoretically these beams do not diffract, but experimental limitations such as finite apertures lead to finite but very long focal lengths^19,22,23^. The detailed discussion of the experimentally achievable invariant length of such beams in Supplementary Note 2 suggests that an enlargement of the size (aperture) of the SLM (for a fixed wavelength *λ*_0_) would have the largest effect on improving the distance of invariant propagation. Figure 2t, u shows a propagation-invariant Bessel-lattice beam ${\psi }_{2,5}^{{\rm{BL}}}$, where the OAM with charge *ℓ* = 2 has the effect of making the square lattice rectangular. An interesting effect can be explored in which the Bessel-lattice beam ${\psi }_{0,5}^{{\rm{BL}}}$ from Fig. 2a–f is made to pass through a *ℓ* = 2 phase vortex plate, resulting in the diffracting field $\psi ={\psi }_{0,5}^{{\rm{BL}}}\cdot \exp \left[{\rm{i}}\ell \phi \right]$. Its experimentally obtained initial intensity and phase distributions are shown in images Fig. 2v, w. A cross-section of the intensity under propagation is shown in Fig. 2x, where we can see that the beam stabilizes during evolution over one Rayleigh length *z*_*e*_ and acquires the shape corresponding to ${\psi }_{2,5}^{{\rm{BL}}}$ as shown in Fig. 2y, z. Hence, the total momentum of the light field is conserved and transforms the initial (diffractive) state to its invariant form when propagating sufficiently far. In Supplementary Notes 1–5, we present further investigations and a generalization of this effect, which can be considered both as angular momentum transfer as well as a form self-healing^2,19,35^.

The first approach described above allows finding the phase functions that produce different caustic shapes. However, a limitation of this approach becomes evident from the example of a cardioid caustic in Fig. 2m–r. Since each point of the Fourier ring contributes to one and only one caustic point, the caustic is either convex or at least the curvature always must have the same sign. That is, inflection points of the desired caustic cannot be included in the intensity distribution, and even regions of small curvature give very faint intensity features as can be appreciated in Fig. 2q. These limitations are overcome by the second approach described in what follows. More information on the algorithm and the experimental setup are given in the ‘Methods.’

### Solving the inverse problem using Bessel beams as a pencil

**Fig. 3:**
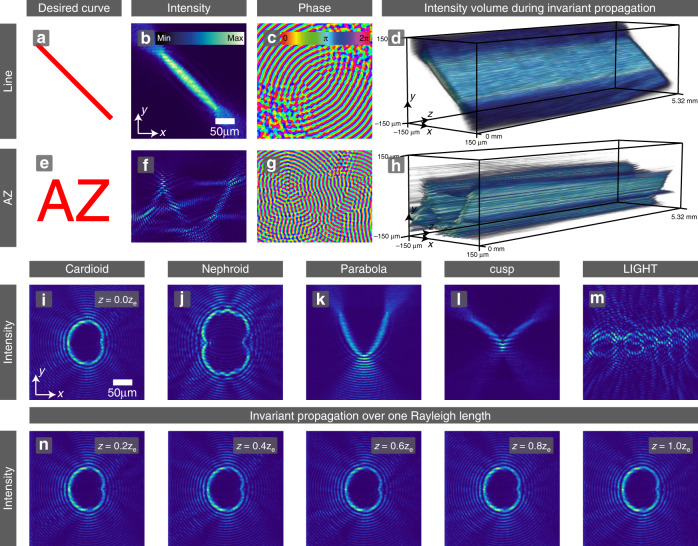
Bessel pencil method for drawing any desired curve. (Top row) Straight line segment. **a** Desired curve. **b**, **c** Experimentally measured transverse intensity and phase. **d** Experimentally obtained intensity volume over one Rayleigh length. (Second row) Superposition of building blocks allows creating cuspoid letters ‘AZ’ in propagation-invariant light. (Third row) Smart beam design facilitates complex structures: cardioid (**i**), nephroid (**j**), parabola (**k**), cusp (**l**) and the word ‘LIGHT’ in propagation-invariant light fields. **n** Proof of invariance during propagation at the example of the cardioid (**i**) over a distance of one Rayleigh length *z*_*e*_.

The second method for producing propagation-invariant intensity features in the transverse plane that follow any desired curve is based on ‘drawing’ this curve using a 0th-order Bessel beam (whose caustic is a point) as a pencil. That is, the desired pattern is generated by superimposing coherently a continuum of 0th-order Bessel beams whose foci trace the anticipated curve. The ‘Methods’ section describes this algorithm in detail. Related approaches to create 3D high-intensity curves based on superimposing Gaussian beams or for lower-dimensional accelerating fields were demonstrated in^36–38^, but without the propagation invariance achieved here. Figure 3 shows a collection of experimentally obtained propagation-invariant beams generated with this algorithm. A propagation-invariant straight line segment, measured over one Rayleigh length *z*_*e*_, is shown in the top row of Fig. 3. By deftly superimposing differently oriented cusps, the propagation-invariant letters ‘AZ’ can be composed over the transverse plane shown in the second row of Fig. 3. The images presented in the third row of Fig. 3 show further propagation-invariant high-intensity rims in the initial transverse plane: a cardioid (i), a nephroid (j), a parabola (k), and a cusp (l) are examples of simple geometric shapes. Notice that the intensity is roughly uniform along the whole curve, regardless of the curvature. Smart beam design even allows for rather complex shapes, demonstrated as a proof of principle by imprinting the word ‘LIGHT’ into the light field, as shown in Fig. 3m. The quality of these complex intensity profiles can be further improved by engineering the interference more rigorously, i.e., by adjusting the relative phase differences of the individual caustic building blocks with respect to each other precisely. The propagation invariance of these and other beams created with the Bessel pencil method is demonstrated for the example of the cardioid from Fig. 3i in the sequence of images in Fig. 3n, whose intensity is preserved over a propagation distance of one Rayleigh length. We show further examples of transverse caustic shapes and their invariance during propagation in Supplementary Note 4.

## Discussion

Caustics can be shaped into propagation-invariant light that presents any desired high-intensity curve in its transverse profile. We present methods to tailor the light’s wavefront, forming translation invariant, customized caustics as the envelope of families of rays. The two approaches presented here are illustrated by experimentally implementing propagation-invariant beams whose caustics trace fundamental forms like astroids, deltoids, cardioids, and nephroids, as well as more sophisticated structures such as letters or words. These self-healing beams propagate robustly in the presence of perturbations. We demonstrate the properties of an astroid-shaped Bessel-lattice beam that interpolates between the sum of four plane waves and a Bessel beam. Propagation-invariant caustics satisfy the need for customized high-energy transfer in nano-fabrication applications with light or electron beams for ultrafast cutting and deep drilling in transparent materials^10,11^. 2D caustic light, e.g., with centered periodic lattices and well-defined curvilinear borders, enables fabricating refractive index modulations in (nonlinear) materials for novel topological structures. Imaging systems like light sheet microscopy benefit from the robust long-focus propagation of high-intensity caustics with phase singularities^14,15^. Similar to the alphabet of orthogonal states of Bessel beams with different order, the presented propagation-invariant caustics can be used to construct other families of orthogonal states with particular self-healing features, thus are highly important for secure high-dimensional (quantum) communication^39,40^.

## Methods

### Experimental setup

**Fig. 4:**
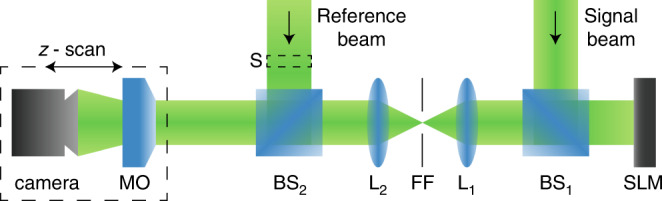
Experimental setup. Nd:YVO_4_ laser source, cw, wavelength *λ*_0_ = 532 nm. SLM spatial light modulator, BS beam splitter, L lens, FF Fourier filter, MO microscope objective, S shutter.

  Figure 4 shows the experimental setup. A frequency-doubled cw Nd:YVO_4_ laser beam with wavelength *λ*_0_ = 532 nm is expanded in a plane wave, illuminating a phase-only full HD SLM ‘Holoeye HEO.’ We address a pre-calculated phase pattern to the SLM that allows encoding the real space amplitude and phase simultaneously on a single phase-only modulator^41^. Therefore, in the common focal plane of the lenses L1 (*f* = 385 mm) and L2 (*f* = 38 mm) an appropriate filter in Fourier space is necessary (FF). The desired light field forms in the image plane of the modulator. A movable microscope objective (MO), Olympus MPLN with a magnification of 10 and numerical aperture of 0.25, together with a camera, iDS UI-3370CP-M-GL, constitute the imaging system, capable of observing the propagation of a light field. A second expanded beam, a plane wave, serves as a reference beam and can be switched on for phase measurements using a shutter (S). To recover the spatial phase, a standard digital holographic method is applied, that is based on the superposition of the signal beam with a slightly tilted reference beam^42^.

### First approach

The caustics, parametrized by *ϕ*, are located at the points **r** for which the first and second derivatives of the phase of the integral in Eq. (1) with respect to *ϕ* vanish:

3 $${\Phi }^{\prime}(\phi )+{k}_{\perp }{\bf{r}}\cdot {{\bf{u}}}^{\prime}(\phi )=0,\quad {\Phi }^{{\prime\prime} }(\phi )-{k}_{\perp }{\bf{r}}\cdot {\bf{u}}(\phi )=0.$$

Since **u** and ${\bf{u}}^{\prime}$ are orthonormal and complete over the plane, we can write ${\bf{r}}=({\bf{r}}\cdot {\bf{u}}){\bf{u}}+({\bf{r}}\cdot {\bf{u}}^{\prime} ){\bf{u}}^{\prime}$ to find the parametrized caustic:

4 $${{\bf{r}}}_{{\rm{c}}}(\phi )=\frac{1}{{k}_{\perp }}\left[{\Phi }^{{\prime\prime} }(\phi ){\bf{u}}(\phi )-{\Phi }^{\prime}(\phi ){{\bf{u}}}^{\prime}(\phi )\right].$$

Note that the angular coordinate *ϕ* in the Fourier ring is not an angular coordinate in the caustic space. A parametrization of the caustic with *ϕ* as the parameter may be found by considering the derivative of both sides of the previous equation:

5 $${{\bf{r}}}_{{\rm{c}}}^{\prime}(\phi )=\frac{1}{{k}_{\perp }}\big[{\Phi }^{\prime\prime\prime }(\phi )+{\Phi }^{\prime}(\phi )\big]{\bf{u}}(\phi ).$$

Cusps in the caustic can occur where ${{\bf{r}}}_{{\rm{c}}}^{\prime}(\phi )=0$, namely, for values of *ϕ* for which ${\Phi }^{\prime\prime\prime }(\phi )+{\Phi }^{\prime}(\phi )=0$. Solving this differential equation allows for the realization of the light field *ψ*(**r**) from Eq. (1) inversely, embedding in it the parametrized caustic **r**(*ϕ*).

### Second approach

A second approach to realize a desired curve is to use the most localized propagation-invariant light spot we can achieve, a 0th-order Bessel beam, as a pencil to draw the curve. Since *ℓ* = 0, its caustic is a point. In accordance with Eq. (1), the angular spectrum for such a Bessel beam centered at **r**_c_ is ${A}_{{\rm{B}}}\exp \left[{\rm{i}}{\gamma }_{{\rm{B}}}-{\rm{i}}{k}_{\perp }{{\bf{r}}}_{{\rm{c}}}\cdot {\bf{u}}(\phi )\right]$. The key to designing the real space light field *ψ*(**r**) is to construct the angular spectrum by coherent integration of this expression along the desired path **r**_c_(*τ*) and choosing the phase *γ*_B_(*τ*) and amplitude *A*_B_(*τ*) appropriately along the curve. Note that the parameter *τ* is not necessarily the azimuthal angle *ϕ*, but the arc length of the curve. Since the phase of the field along the curve should grow with the curve’s arc length *τ*, it is given as:

6 $${\gamma }_{{\rm{B}}}(\tau )={k}_{\perp } \int_{0}^{\tau }\left|{{\bf{r}}}_{{\rm{c}}}^{\prime}(s)\right|{\rm{d}}s.$$

Similarly, to make the weight uniform along the curve we use:

7 $${A}_{{\rm{B}}}(\tau )\propto 1/\sqrt{\left|{{\bf{r}}}_{c}^{\prime}(\tau )\right|}.$$

The total light field that contains a high-intensity curve **r**_c_(*τ*), parametrized by *τ*, between two points *A* and *B* can then be calculated via the angular spectrum as:

8 $$\psi ({\bf{r}})= \int _{A}^{B}{A}_{{\rm{B}}}(\tau )\exp \left[{\rm{i}}{\gamma }_{{\rm{B}}}(\tau )-{\rm{i}}{k}_{\perp }{{\bf{r}}}_{{\rm{c}}}(\tau )\cdot {\bf{u}}(\phi )\right]{\rm{d}}\tau .$$

## Supplementary information

Supplementary Information

Description of Additional Supplementary files

Supplementary Movie 1

Supplementary Movie 2

Supplementary Movie 3

## Data Availability

The datasets generated during and/or analyzed during the current study are archived in the AP-WWU repository and are available from the corresponding author on reasonable request. Source data are provided with this paper.

## Source data

Source Data
