# Supplementary material for: Shaping caustics into propagation-invariant light
Source: Nat Commun. 2020 Jul 17;11:3597. doi: 10.1038/s41467-020-17439-3 (PMC7367875; doi:10.1038/s41467-020-17439-3)
Supplement: Supplementary file 1 — Supplementary Information [file 41467_2020_17439_MOESM1_ESM.pdf]

Shaping caustics into propagation-invariant light — Supplementary Information

Alessandro Zannotti* and Cornelia Denz
Institute of Applied Physics and Center for Nonlinear Science (CeNoS), University of Muenster, 48149 Muenster, Germany

Miguel A. Alonso
Aix Marseille Univ, CNRS, Centrale Marseille, Institut Fresnel, UMR 7249, 13013 Marseille, France,
The Institute of Optics, University of Rochester, Rochester NY 14627, USA

Mark R. Dennis
School of Physics and Astronomy, University of Birmingham, Birmingham B15 2TT, UK
H H Wills Physics Laboratory, University of Bristol, Bristol BS8 1TL, UK
(Dated: May 15, 2020)

SUPPLEMENTARY NOTE 1: VISUALIZING THE RAYS OF PROPAGATION INVARIANT CAUSTICS

We assume that the beams with propagation-invariant caustics propagate in linear, homogeneous, and isotropic media, so that their rays are straight lines. Caustics are defined as the envelopes of these rays [1, 2]. Each ray is described by its transverse coordinate $\mathbf{Q} = (Q_x, Q_y)$ in the initial plane $z = 0$ and its transverse momentum $\mathbf{P} = (P_x, P_y)$. The momentum is determined from the derivative of the 1D phase function $\Phi(\phi)$ mapped onto the Fourier ring in cylindrical coordinates. The evolution in z of one ray is given by $\mathbf{Q} + z\mathbf{P}$ [3].

For Bessel beams with topological charge $l \neq 0$ the envelope of the rays describes a circular caustic, as shown in Fig. 1(a) in the main part of this paper [3]. The rays of the astroid beam form an envelope that is characterized by four cusps, shown in Fig. 1 & 2 in the main manuscript. The left site of Supplementary Figure 1 shows the same subset of rays from several viewpoints, in order to show that the rays in the transverse plane emerge as a mapping from the three-dimensional rays to the plane. Recall that the full ray family is composed of a continuum of versions of the rays shown here displaced in the z-direction. This is demonstrated on the right site of Supplementary Figure 1, where we add for illustration purposes 5 z-shifted subsets of rays (blue), forming an invariant caustic (red). In each iteration, the additional subset is highlighted by bold lines. The 5 discrete sets should give an impression of the continuous ray picture. An animation of the rotation through the 3D subset ray volume can be downloaded here (Supplementary Movie 1), as well as the visualization of the assembly of the full ray picture and its caustic (Supplementary Movie 2).

* a.zannotti@uni-muenster.de

SUPPLEMENTARY NOTE 2: ESTIMATION OF THE INVARIANT PROPAGATION LENGTH

Theoretically, beams that obey Eq. (1) of the main manuscript do not change their transverse intensity distribution, independently of any propagation distance z. However, experimental limitations like finite transverse sizes of the beams corresponding to the SLM dimensions and their imaging lenses naturally lead to finite but still comparatively long longitudinal distances (several Rayleigh lengths) in which the beams can be considered as being propagation invariant, by keeping, in good approximation, the original transverse intensity distribution $I_0(\mathbf{r}_\perp)$ at $z = 0\,\mathrm{mm}$ [4].

This concept is visualized schematically in Supplementary Figure 2(a) at the example of two finite plane waves with wave vectors $k_1 = k_\perp$ and $k_2 = -k_\perp$, located on the Fourier space ring, that interfere in the far field (the real space) and form intensity fringes $I(y) \propto \cos^2(k_\perp y)$. The volume in which invariant fringes occur is finite, and as shown here it has a rhombus cross-section. Its dimensions depend on the transverse size g of the finite wave contributions (due to apertures in the experimental setup) and the angle $\theta = \sin^{-1}(k_\perp/k_0) = \sin^{-1}(\lambda_0/a) \approx 2^\circ$ (cf. Sec. *Caustics in propagation-invariant beams*), thus in turn on the light's wavelength $\lambda_0 = 532\,\mathrm{nm}$ and the characteristic real space structure size $a = 2\pi/k_\perp = 15\,\mu\mathrm{m}$.

In order to estimate the quality of our beams in terms of the length in which such beams can be considered to have an invariant transverse intensity profile, we investigate the propagation of a Bessel-lattice beam as an example. Note that the quality depends in parts on the individual experimental realization and the particular setup. Supplementary Figure 2(b) shows a yz cross-section through the intensity volume, obtained by measuring 397 transverse intensity distributions over a range of 40 mm, symmetrically around the origin at $z = 0\,\mathrm{mm}$. As one Rayleigh length of this beam is $z_e = 2k_0a^2 = 5.31\,\mathrm{mm}$, the length of 40 mm corresponds to 7.5 Rayleigh lengths. The five transverse intensity distributions below are obtained at the longitudinal positions indicated

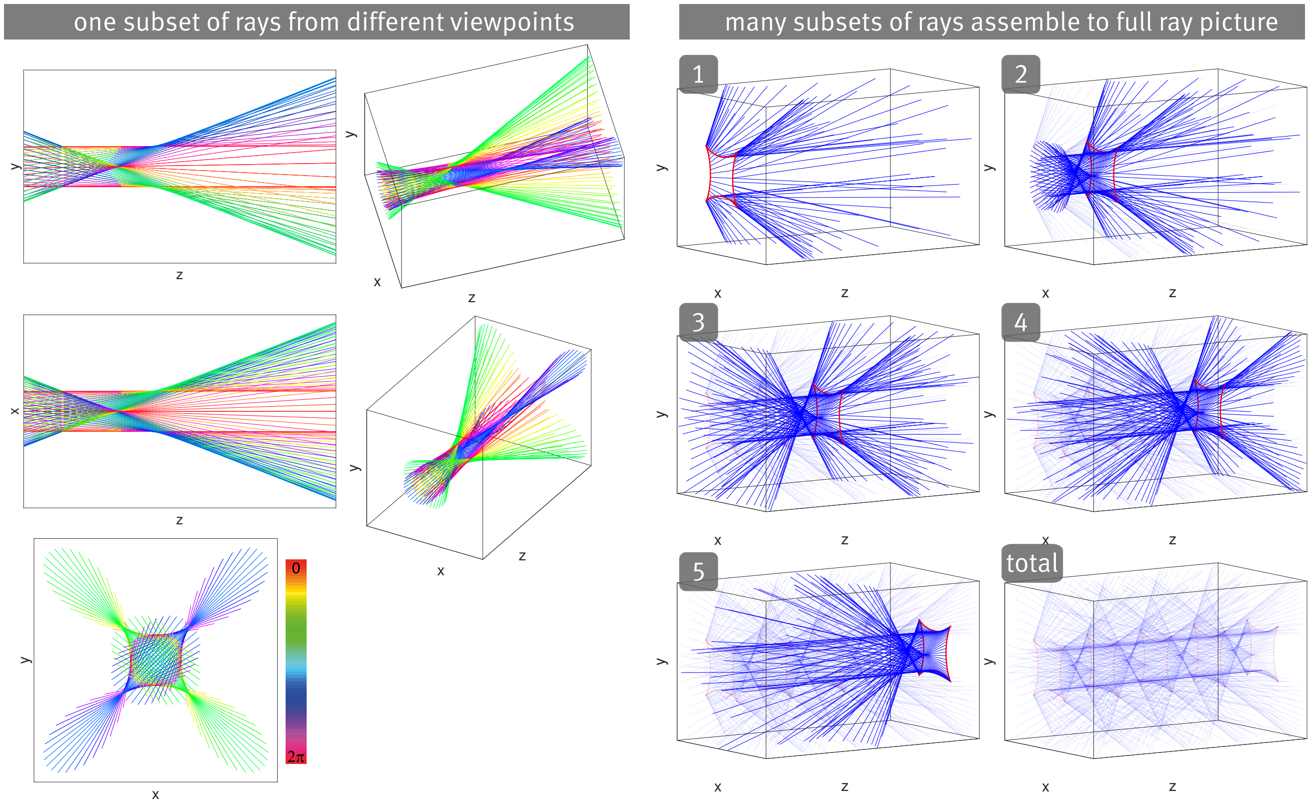

Supplementary Figure 1. Left: One subset of the ray picture of a tailored propagation-invariant astroid caustic. Color corresponds to phase. Shown are different angles of view to illustrate how this subset of the family of rays forms the caustic. Right: Assembly of the whole ray picture (total) by adding a continuum (five of them shown here) of z-shifted subsets of rays together. Rays are blue, caustics are red.

in the yz cross-section by red lines. They show the transition from the best experimentally realizable state at $z = 0\,\mathrm{mm}$, to which we refer as $I_0(\mathbf{r}_\perp)$, to states that propagated so far that the pattern is beyond the invariant area ($|z| > 11.5\,\mathrm{mm}$). In order to highlight the darker areas in the yz cross-sections, for visual convenience we show the square root of the (normalized) intensity. This visualization allows to recognize the rhombus shape of the diffracting beam.

The rhombus shape is estimated and indicated with red dashed lines in Supplementary Figure 2(c). The aim of this evaluation is to get a qualitative explanation for the finite length of the area with invariant intensity. From this sketch, we estimate that the lengths of the two diagonals of the rhombus are $A = (23 \pm 5)\,\mathrm{mm}$ and $B = (820 \pm 25)\,\mathrm{\mu m}$, hence its height can be calculated to be $g = AB/\sqrt{A^2 + B^2} = (819.5 \pm 24.9)\,\mathrm{\mu m}$. The size g corresponds to the dimension of the contributing (theoretically infinitely) extended plane waves that form the Bessel-lattice beam. From our estimation, we calculate an opening angle of $\theta = \sin^{-1}(2AB/(A^2 + B^2))/2 \approx (2.0 \pm 0.4)^\circ$, which is in agreement with the previously stated opening angle.

As a consequence of a finite wave expansion g, the Fourier ring is not infinitesimal thin, but has a size of $2\pi/g$. Supplementary Figure 2(d) compares, true to scale, the radius of the Fourier ring $2\pi/a$, where $a = 15\,\mathrm{\mu m}$, to the thickness of the ring. The image shows the calculated Fourier space intensity distribution. From this, we see that the ring still is comparatively thin and thus ensures several Rayleigh lengths in which the beam can be considered as propagation invariant. Further, taking into account the physical size of the SLM $L_{\mathrm{SLM}} = 15.36\,\mathrm{mm}$, imaged by the telescope L_1-L_2 with a demagnification of $M = 9.4$, gives a corresponding feature size of $g = L_{\mathrm{SLM}}/2/M \approx 820\,\mathrm{\mu m}$.

A good measure for the similarity of the original transverse intensity distribution $I_0(\mathbf{r}_\perp)$ at $z = 0\,\mathrm{mm}$ with transverse intensity distributions $I(\mathbf{r}_\perp, z)$ at any longitudinal position z is the normalized cross-correlation function (denoted by $\star$)

$$\gamma\left(\mathbf{r}_\perp\right) = \frac{\left[I_0 \star I(z)\right]\left(\mathbf{r}_\perp\right)}{\sigma_{I0}\sigma_{Iz}}, \qquad (1)$$

where σ_{I0} and σ_{Iz} are standard deviations of the respective intensities [5].

For the experimentally obtained 400 transverse intensity distributions, we calculate the z-dependent normalized cross-correlation $\gamma\left(\mathbf{r}_\perp\right)$ and show its respective maximum value in Supplementary Figure 2(e). Around the

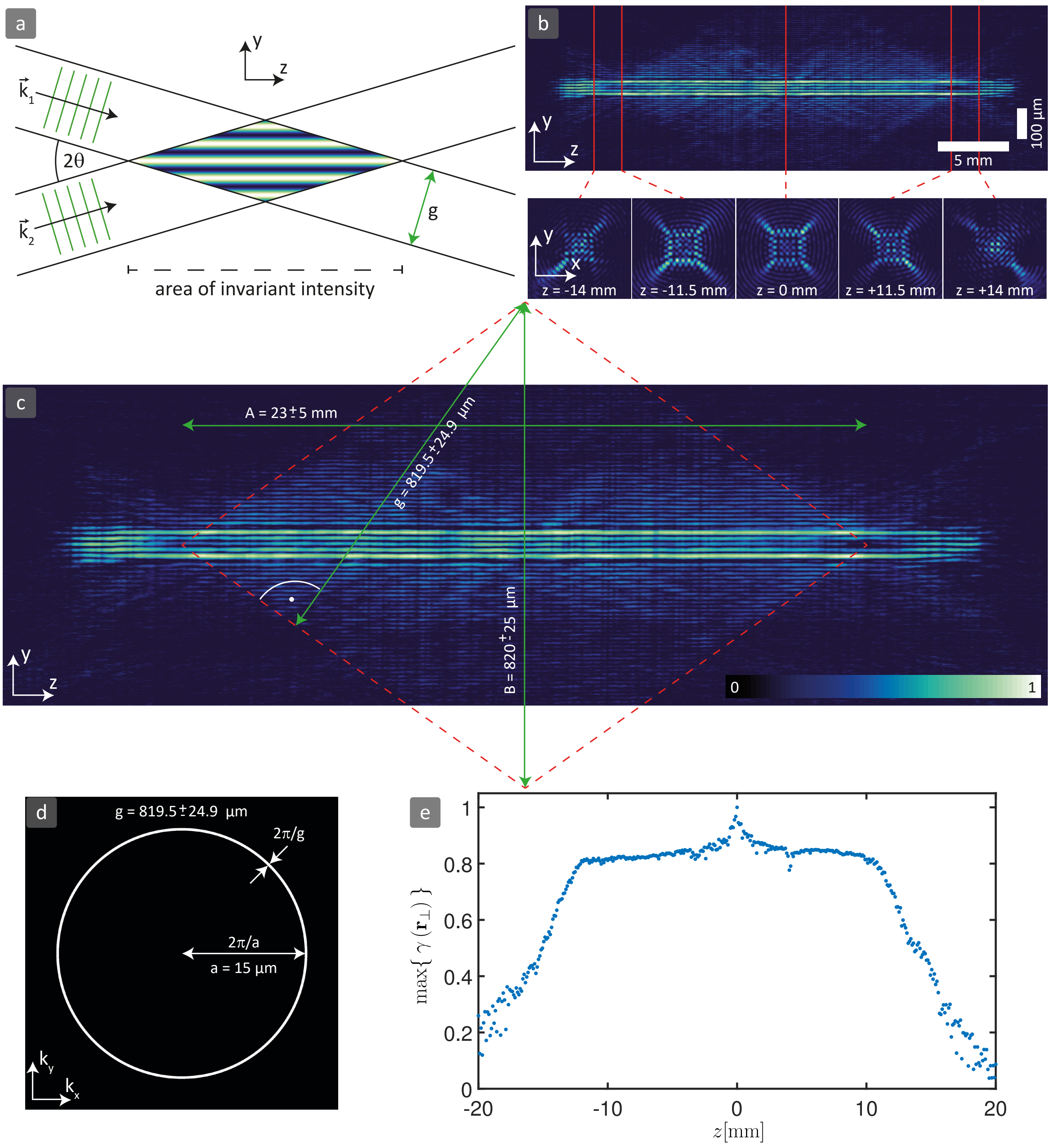

Supplementary Figure 2. Quality of the intensity invariance during propagation at the example of the Bessel-lattice beam. (a) Sketch of the formation of an rhomboid area in which the intensity is invariant. (b,c) Experimentally measured yz cross-section through the intensity volume and estimation of the dimensions of the rhomboid area. (d) True to scale presentation of the Fourier space intensity ring with particular emphasis on the ratio of its radius to its thickness. (e) Maximum of the normalized cross-correlation γ in dependence of the propagation distance z. The reference is the plane at $z = 0\,\mathrm{mm}$. Source data are provided as a Source Data file.

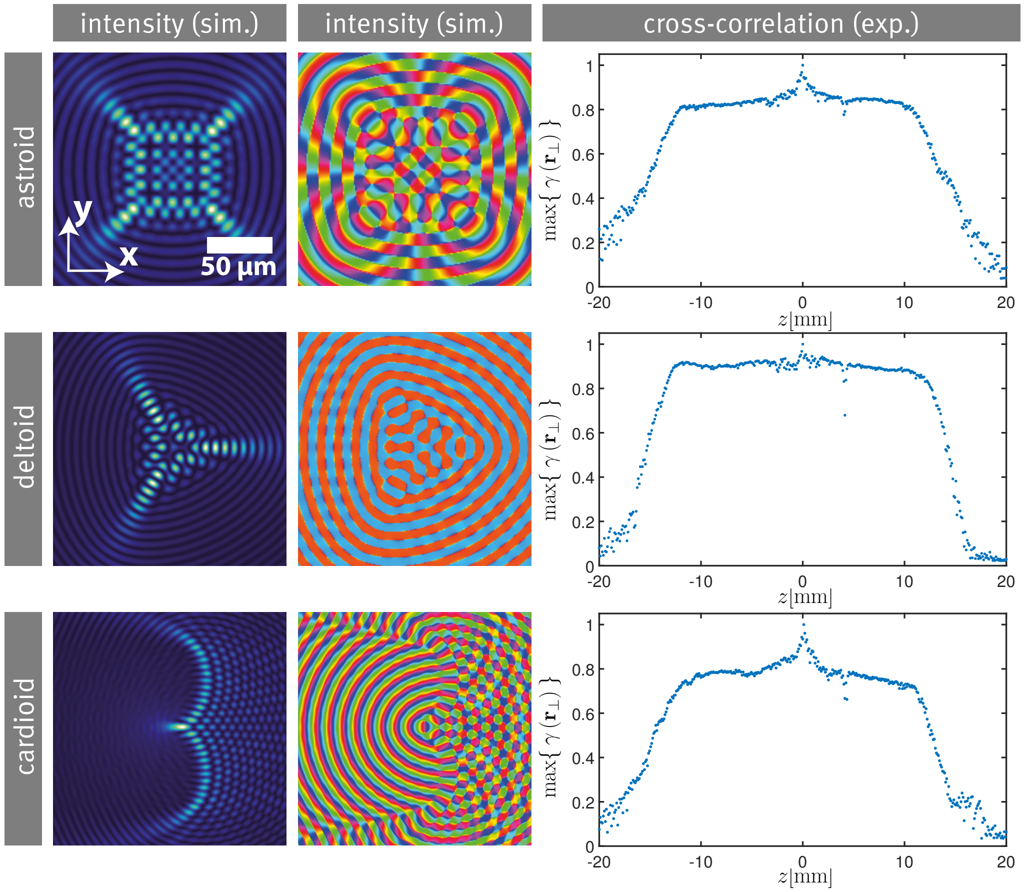

Supplementary Figure 3. Cross-correlations as a demonstration of the invariance of the astroid, deltoid, and cardioid. The transverse intensity and phase distributions represent simulations in order to be compared with the experimentally obtained distributions ins Fig. 2. The cross-correlation graphs are obtained from experimental data. Source data are provided as a Source Data file.

origin at $z = 0\,\mathrm{mm}$, the cross-correlation achieves its maximal value of unity. Around the origin, a plateau is apparent, with correlation values of more than 80%, symmetrically extended over more than 20 mm. In this area, the realized Bessel-lattice beam has, in very good approximation, an invariant transverse intensity pattern. Beyond these distances, diffraction influences the beam center and the degree of the cross-correlation decreases.

Similar results are existing for all presented propagation-invariant beams, as they obey the same construction rules and are realized in the same experimental system. The beams of Fig. 2 are shown as simulated intensity and phase distributions in Supplementary Figure 3(with a very good agreement). They have comparable, experimentally obtained cross-correlation signatures. The extended plateaus around $z = 0\,\mathrm{mm}$ prove that these beams propagate invariantly for several Rayleigh lengths. Further examples of propagation-invariant beams and the evaluation of their invariance are shown also in Supplementary Figure 6.

The analysis presented in this section shows that the length of the invariant volume can be further enhanced using a larger SLM, a smaller wavelength λ_0, or a larger characteristic structure size a. The implementation of these enhancements is important for advanced imaging applications with a range of distances typically used in microscopy with ultra-long focal depths.

SUPPLEMENTARY NOTE 3: GENERALIZED MOMENTUM TRANSFER IN PROPAGATION INVARIANT CAUSTIC BEAMS

We discuss how a propagation-invariant beam can be made to morph into another by making it pass through a transparent mask with the appropriate azimuthally-dependent phase. Consider first the example of a propagation-invariant Bessel-lattice beam $\psi^{\mathrm{BL}}_{\ell,q}(x,y)$ shown in Fig. 2. As shown there, by modifying its phase with a vortex mask of charge $m = 2$, the field acquires an intensity pattern that changes under propagation until it settles (for $z > z_e$) into the propagation-invariant field (at least in the center) of a corresponding Bessel-lattice beam with changed topological charge $\psi^{\mathrm{BL}}_{\ell+m,q}(x,y)$. This

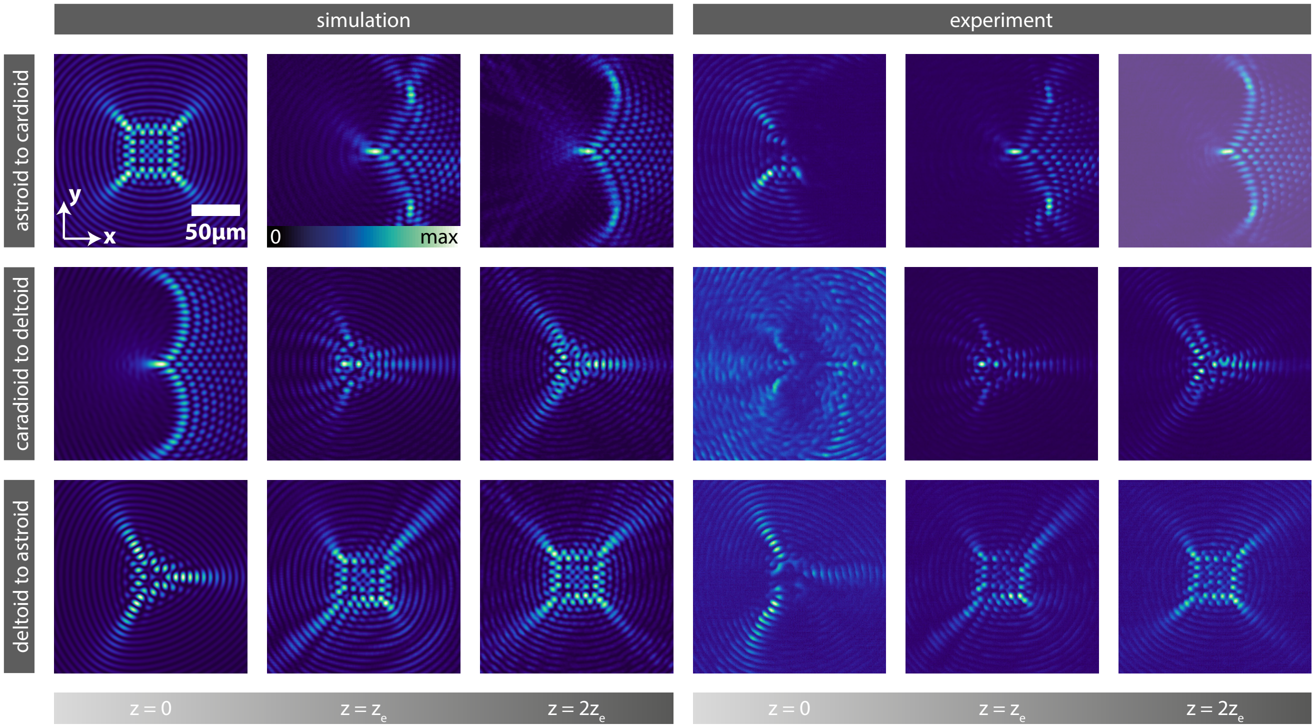

Supplementary Figure 4. Momentum transfers of an initial caustic to a final caustic by tailoring the initial phase. Compared are simulations with experiments. During propagation, an astroid transforms to a cardioid caustic (top), a cardioid transforms to a deltoid caustic (middle), and a deltoid transforms back to an astroid caustic (bottom). The propagation distances are 1 and 2 Rayleigh lengths z_e.

process is represented symbolically as

$$\lim_{z\to\infty} \psi^{\mathrm{BL}}_{\ell,q}(x,y,z)\cdot \exp\left[\mathrm{i}m\theta\right] \to \psi^{\mathrm{BL}}_{\ell+m,q}(x,y), \qquad (2)$$

where θ is the azimuthal angle.

We generalize this behaviour to any initial and final propagation-invariant beams, ψ^i and ψ^f, whose phase functions are given by $\Phi^i(\theta)$ and $\Phi^f(\theta)$, respectively. The transition can be then implemented by using a transparent mask whose phase is $\Phi^f(\theta) - \Phi^i(\theta)$, namely

$$\lim_{z\to\infty} \psi^i \cdot \exp\left[\mathrm{i}\left(\Phi^f - \Phi^i\right)\right] \to \psi^f. \qquad (3)$$

We demonstrate this effect by transferring the three fundamental caustic shapes from Fig. 2, the astroid, the cardioid and the deltoid, into each other during propagation, shown in Supplementary Figure 4. The very left columns show simulated initial transverse intensity distributions of the corresponding electric fields, whose individual phases are manipulated. During propagation, the transverse intensity distributions change. After one Rayleigh length z_e the final shapes are apparent as shown in the second column of Supplementary Figure 4, and fully developed after two Rayleigh lengths, as shown in the third column. The experimental results on the right half of Supplementary Figure 4 agree with the simulations. Only the initial intensity distributions show deviations from the simulations since the spatial light modulator is not capable to realize all of the (spatial) high-frequencies of the phase distribution that fluctuates strongly and interferes destructively. A video that shows the propagation of these three beams in simulation and experiment can be found here (Supplementary Movie 3).

SUPPLEMENTARY NOTE 4: BESSEL PENCIL FOR COMPLEX HIGH-INTENSITY FEATURES IN PROPAGATION-INVARIANT LIGHT

This section provides more experimental results and shows sophisticated high-intensity distributions realized with the *Bessel pencil method*. We show the spectra of the beams in Fig. 3, present several further examples whose high intensity curves describe both fundamental and rather complex shapes, and demonstrate their invariant propagation.

In order to create desired propagation-invariant high-intensity curves in the transverse plane, we integrate 0th-order Bessel beams, whose caustics are points [3], coherently along these paths. We modulate both the amplitude and phase of the spectrum, which is confined on a ring. Supplementary Figure 5 shows the calculated intensity and phase distributions of the spectra and the corresponding propagation-invariant experimentally obtained transverse intensity and phase distributions.

All images in Supplementary Figure 6 are obtained experimentally. The initial transverse intensity distribu-

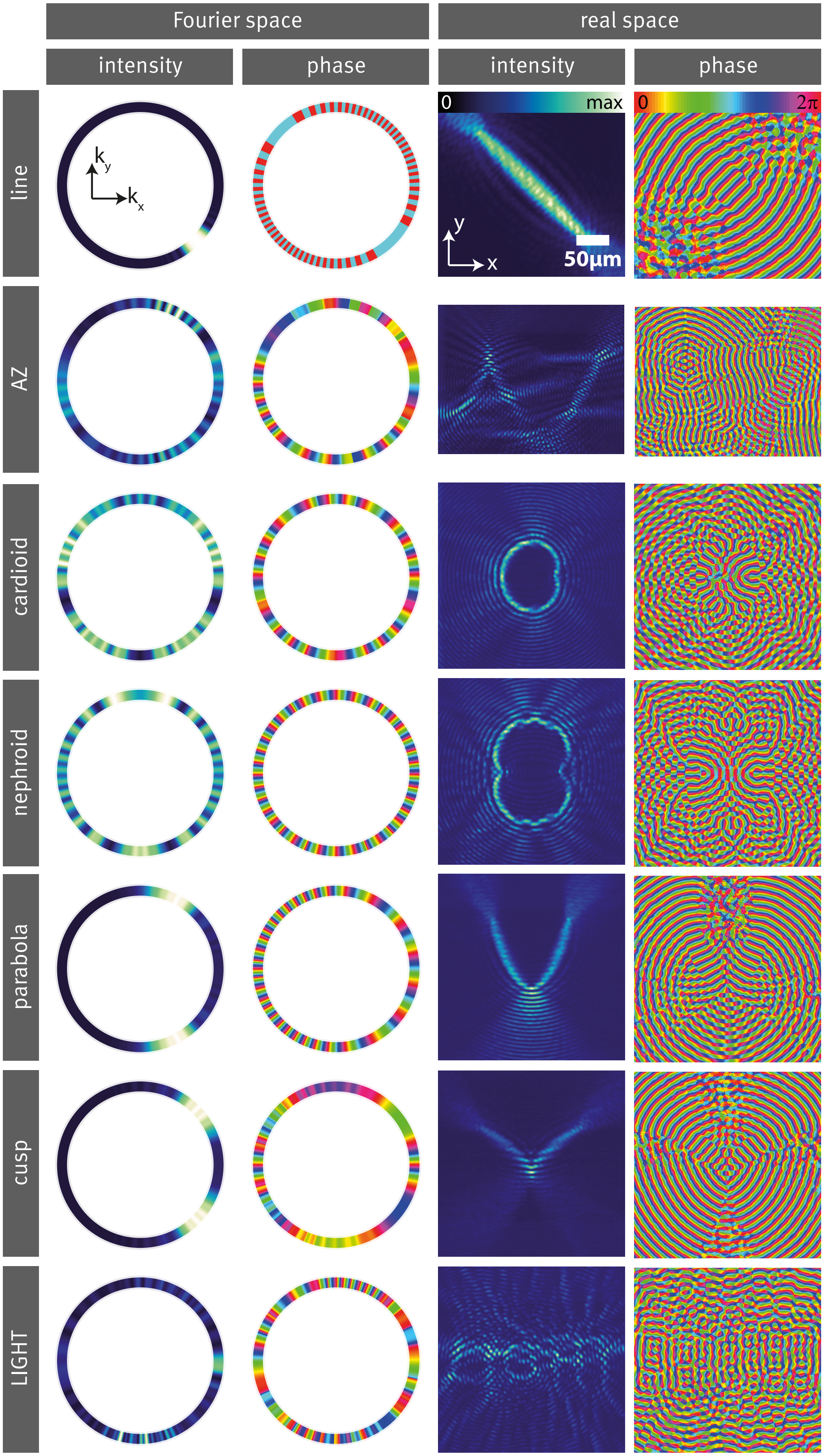

Supplementary Figure 5. Relation between the Fourier space intensity and phase distributions on a ring and the corresponding real space transverse intensities and phases for different curves realized with the *Bessel pencil method*.

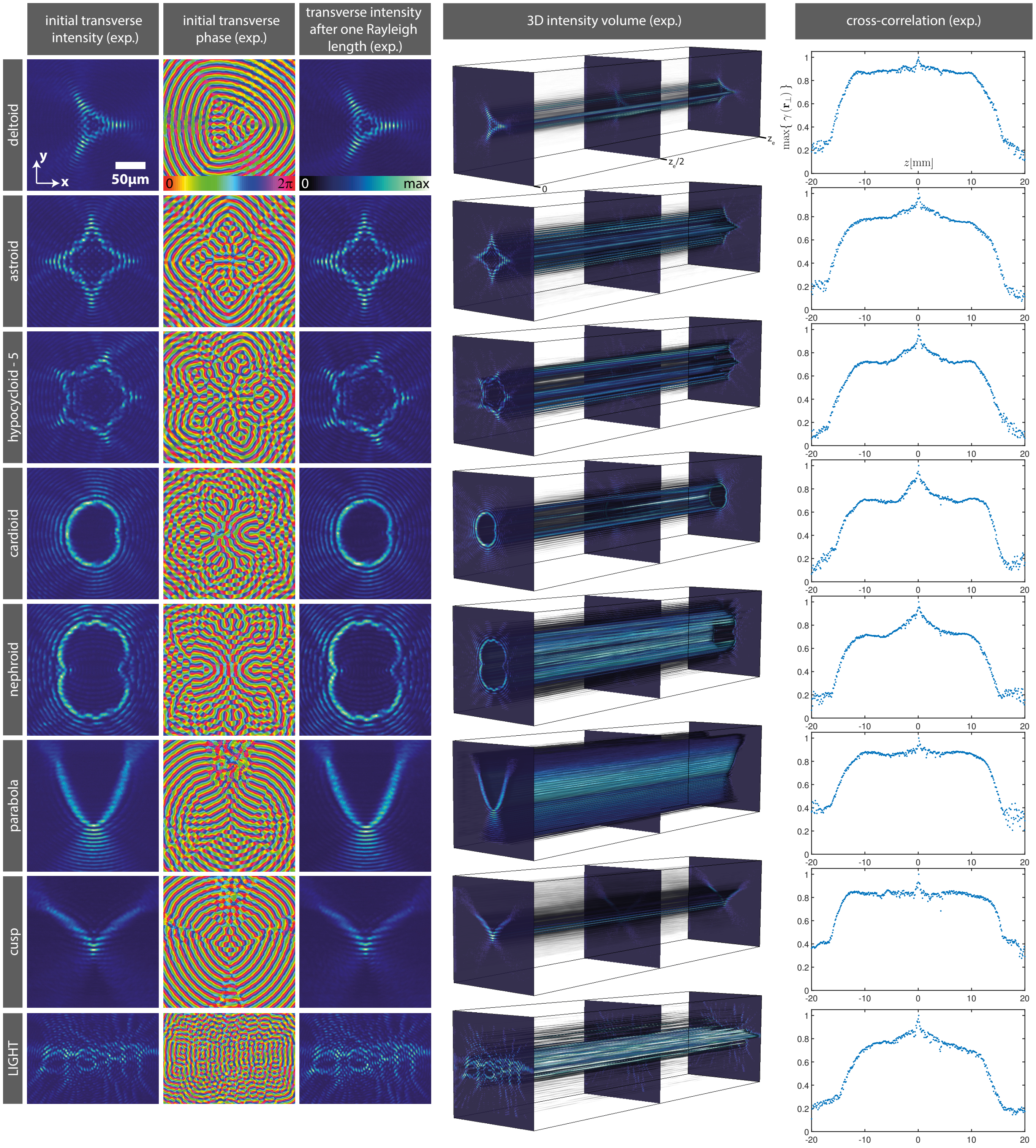

Supplementary Figure 6. A collection of experimentally-realized propagation-invariant beams realized with the *Bessel pencil method*, whose profiles trace the following shapes: deltoid, astroid, hypocycloid with 5 cusps, cardioid, nephroid, parabola, cusp, and letters forming the word LIGHT. The initial transverse intensity and phase distributions are shown in columns 1 & 2, respectively. Column 3 shows the (largely unchanged) transverse intensity after propagating one Rayleigh length. Column 4 shows the experimentally obtained 3D intensity volume. Column 5 shows the maximum values of the cross-correlation of the intensity at the imaging plane with the plane at varying z positions. Source data are provided as a Source Data file.

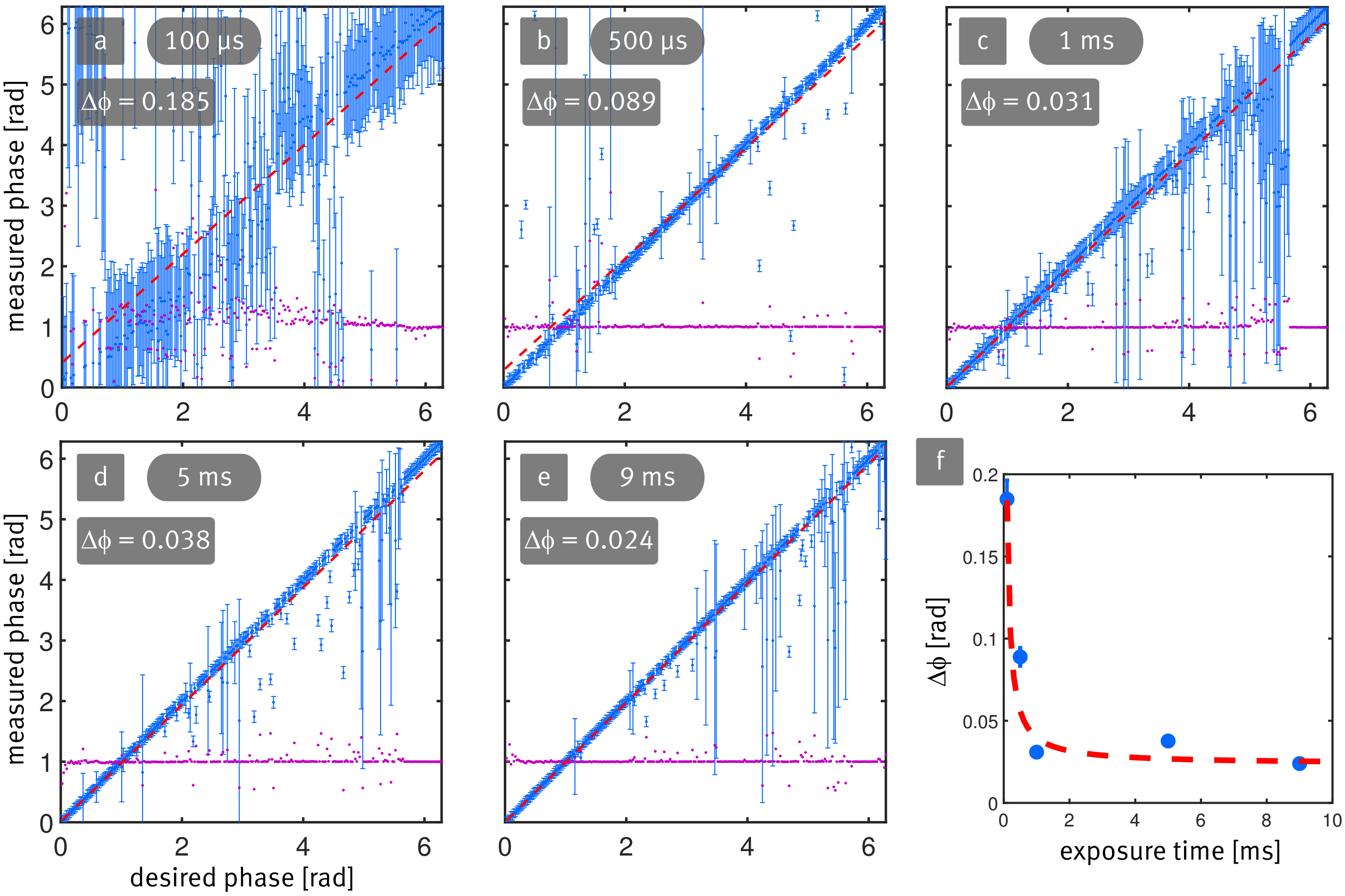

Supplementary Figure 7. Accuracy of the phase measurements as a function of the exposure time. (a) Measured phase as a function of the desired phase. The mean of the measured spatial phase and its standard deviation are shown in blue. The ratio of the desired and measured phases is shown in purple. The linear fit is shown as a red dashed line and its uncertainty $\Delta\phi$ is given in the insets. (b) Measured uncertainties of the phases $\Delta\phi$ as a function of the exposure time. Source data are provided as a Source Data file.

tions can be seen in column 1, while column 2 shows the corresponding phases. The transverse intensity distributions after propagating one Rayleigh length $z_e = 5.32\,\mathrm{mm}$ are shown in column 3. Column 4 shows 3D intensity volumes created from taking 50 transverse intensity distributions in equidistant steps from $z = 0\,\mathrm{mm}$ to $z = z_e = 5.32\,\mathrm{mm}$.

We demonstrate a deltoid (row 1), and astroid (row 2), which are hypocycloids with 3 or 4 cusps, respectively. They are followed by a hypocycloid with 5 cusps (row 3). From the class of epicycloids, we realized the cardioid (row 4) and the nephroid (row 5). Further, a parabola (row 6) and a cusp (row 7) may act as building blocks for rather complex shapes, such as words. For example, we create the letters LIGHT in a propagation invariant light field, demonstrating the potential of our approach (row 8).

SUPPLEMENTARY NOTE 5: ACCURACY OF THE (PHASE) MEASUREMENTS

In this section we provide some details regarding the uncertainties in the experimental results. The dynamic range of the camera corresponds to 12 bits. Experimentally, we verified the linearity of the detected intensity (gray values) of the camera. The goodness of the linear fit is characterized by a value of $R^2 = 0.99962$. The discretization of the generated intensity values is limited by the 256 gray values that can be imprinted by the SLM.

Using a digital holographic method [6], we perform a phase measurement by superimposing the signal beam with a slightly tilted plane wave. The Fourier transform of the recorded fringe pattern shows the 0th, 1st and -1st diffraction order. Both higher orders contain the full information of the complex field, so that a bandpass filter is applied followed by an inverse Fourier transform of one of these higher orders. We correct all measured transverse spatial phase distributions with a reference phase, obtained by interfering a plane wave as signal beam with

the tilted plane wave.

In order to characterize the accuracy of the phase measurement in our system, we measure the phase difference of a reference area on the SLM with respect to a second area where we increase the phase value gradually and compare the result to the desired phase, as shown in Supplementary Figure 7. This is done for a constant laser power as a function of the exposure time of the camera, as the error increases for low intensities, which is related to the decrease of the detectability of the interference fringes and their declining contrast. Therefore, we obtain the mean (blue values) and the corresponding standard deviation (error bars) of the measured (spatial) phase value distribution over the 256 steps. The ratio of the desired phase to the measured phase should ideally be 1 and is shown in purple. Each set of measured values is fitted with a line. The goodness of the fit is stated in each sub-image and summarized in image (b), which shows the phase uncertainty $\Delta\phi$ as a function of the exposure time. We indicate the trend of the distribution of these uncertainties with the dashed red line, which follows a hyperbolic trajectory. The deviation of the measured values from a line reduces with increasing exposure time and converges asymptotically to the minimum uncertainty of our measurement system given by $\Delta\phi \approx 0.024\,\mathrm{rad}$. For the cases presented in our work, where we use typical exposure times of 5-10 ms at the same constant laser power as for these measurements shown here, we can assume an error of the phase measurement in the order of $\Delta\phi \leq 0.05\,\mathrm{rad}$.

Supplementary References

[1] M. V. Berry and C. Upstill, Prog. in Opt. **18**, 257 (1980).

[2] R. Blandford and R. Narayan, Astrophysical Journal **310**, 568 (1986).

[3] M. A. Alonso and M. R. Dennis, Optica **4**, 476 (2017).

[4] Z. Bouchal, Czechoslovak Journal of Physics **53**, 537 (2003).

[5] L. P. Lewis, "Fast Normalized Cross-Correlation,".

[6] U. Schnars and W. Jueptner, *Digital Holography* (Springer, Berlin, Heidelberg, New York, 2005).
