# Supplementary material for: Shaping caustics into propagation-invariant light
Source: Nat Commun. 2020 Jul 17;11:3597. doi: 10.1038/s41467-020-17439-3 (PMC7367875; doi:10.1038/s41467-020-17439-3)
Supplement: Supplementary file 2 — Description of Additional Supplementary files [file 41467_2020_17439_MOESM2_ESM.pdf]

Description of Additional Supplementary Files

File name: Supplementary Movie 1

Description: One subset of the ray picture of a tailored propagation-invariant astroid caustic. Colour corresponds to phase. Shown are different angles of view to illustrate how this subset of the family of rays forms the caustic.

File name: Supplementary Movie 2

Description: Assembly of the whole ray picture (total) by adding a continuum (five of them shown here) of z-shifted subsets of rays together. Rays are blue, caustics are red.

File name: Supplementary Movie 3

Description: Momentum transfers of an initial caustic to a final caustic by tailoring the initial phase. Compared are simulations (top) with experiments (bottom). During propagation, an astroid transforms to a cardioid caustic (left), a cardioid transforms to a deltoid caustic (middle), and a deltoid transforms back to an astroid caustic (right). The propagation distances are 1 and 2 Rayleigh lengths z_e.
